# The dual-function of HSP70 in immune response and tumor immunity: from molecular regulation to therapeutic innovations

**DOI:** 10.3389/fimmu.2025.1587414

**Published:** 2025-04-14

**Authors:** Beining Zhang, Ruiqun Qi

**Affiliations:** ^1^ Health Sciences Institute, China Medical University, Shenyang, Liaoning, China; ^2^ Department of Dermatology, The First Hospital of China Medical University, Shenyang, China; ^3^ Key Laboratory of Immunodermatology, Ministry of Education, and National Health Commission; National Joint Engineering Research Center for Theranostics of Immunological Skin Diseases, Shenyang, China

**Keywords:** HSP70, immune regulation, tumor immunity, molecular mechanisms, therapeutic strategies

## Abstract

Heat shock protein 70 (HSP70) is a highly conserved molecular chaperone that plays a core role in assisting protein folding and maintaining cellular homeostasis. In recent years, studies have revealed that HSP70 has dual functions in immune regulation: on the one hand, it enhances immune responses by activating non-specific immunity (such as Toll-like receptor 2/4 (TLR2/4) signaling pathways) and specific immunity (such as cross-presentation of antigens, T helper 1 (Th1)/T helper 17 (Th17) differentiation); on the other hand, it inhibits excessive immune reactions by inducing the differentiation of regulatory T cells (Treg) and promoting the secretion of anti-inflammatory factors [such as interleukin-10 (IL-10)]. In cancer, the duality of HSP70 is also very prominent: it can drive tumor progression through pathways such as inhibiting apoptosis, promoting angiogenesis, and tumor metastasis, and it can also inhibit tumor growth by activating immunogenic cell death (ICD), enhancing antigen presentation, and natural killer (NK) cell activity. This review aims to systematically analyze the immune regulatory functions of HSP70, focusing on its dual regulatory mechanisms and the “double-edged sword” nature of HSP70 in tumor immunotherapy and the innovative nature of targeted strategies, as well as providing a theoretical basis and research directions for precision medicine in the treatment strategies of related diseases.

## 1 Introduction

The heat shock protein family (HSPs), as a group of highly conserved molecular chaperones, exhibits remarkable conservation throughout biological evolution (1–4). Studies have shown that HSPs are expressed in a wide range of organisms, from prokaryotes to eukaryotes, including bacteria and humans (1, 3, 5). HSPs play a crucial role in maintaining cellular protein homeostasis under both physiological and stress conditions. Their functions are mainly reflected in two aspects: one is to assist the correct folding of newly synthesized proteins, and the other is to prevent the aggregation of misfolded proteins (6–8), thus effectively avoiding cellular damage (5).

Based on the differences in molecular weight, HSPs can be divided into several subfamilies, including HSP100, HSP90, HSP70, HSP60, and small HSPs. Among them, HSP70 (70 kDa heat shock protein) has attracted much attention due to its multiple functions in protein folding, degradation, and stress adaptation (9). This protein can be induced by a variety of stress factors, including heat shock, oxidative stress, inflammation, and hypoxia (10). Inside the cell, HSP70 mainly functions as a molecular chaperone (11–13). Its main mode of action is to bind to the exposed hydrophobic regions of nascent or misfolded polypeptides, using ATP-dependent conformational changes to promote the correct folding of proteins (14)and prevent the aggregation of misfolded proteins (15, 16). The roles of HSP70 in non-tumor diseases and physiological processes are also remarkable. For instance, in neurodegenerative diseases like Alzheimer’s and Parkinson’s, HSP70 promotes the clearance of misfolded proteins (17–19). In cardiovascular diseases, HSP70 also mitigates myocardial ischemia-reperfusion injury by stabilizing mitochondrial integrity (20).

It is worth noting that in addition to its function in molecular chaperone, non-tumor diseases and physiological processes, studies have also found that under specific conditions, HSP70 can participate in the immune regulation process.

The immune system, as the body’s defense mechanism, consists of two main parts: non-specific immunity and specific immunity. Non-specific immunity rapidly recognizes pathogen-associated molecular patterns (PAMPs) and damage-associated molecular patterns (DAMPs) through pattern recognition receptors (PRRs), thereby activating inflammatory responses and initiating adaptive immune responses (21–23). Specific immunity, on the other hand, establishes long-term immune memory through antigen-specific responses of T lymphocytes and B lymphocytes. The synergistic effect of these two immune mechanisms can not only effectively resist pathogen infections but also eliminate abnormal cells (such as tumor cells) and avoid self-tissue damage through immune tolerance mechanisms. However, dysfunction of the immune system can lead to a variety of diseases. For example, excessive activation may trigger autoimmune diseases, while immune suppression may promote tumor immune escape (24, 25).

Research has found that HSP70 exhibits dual roles in immune regulation: on the one hand, it can enhance antigen presentation efficiency and activate non-specific immune responses (25–27); on the other hand, it can also exert immune suppressive effects by promoting the differentiation of regulatory T cells (Treg) (28, 29) and inhibiting pro-inflammatory cytokines (30, 31). This dual regulatory characteristic is particularly prominent in tumor immunity: HSP70 can both promote anti-tumor immune responses (through cross-presentation of tumor antigens) and support tumor progression by facilitating immune escape and angiogenesis.

This review aims to systematically analyze the immune regulatory functions of HSP70, focusing on its dual regulatory mechanisms and the “double-edged sword” nature of HSP70 in tumor immunotherapy and the innovative nature of targeted strategies, as well as providing a theoretical basis and research directions for precision medicine in the treatment strategies of related diseases.

## 2 Molecular structure and canonical functions of HSP70

### 2.1 Molecular structural characteristics of HSP70

HSP70 comprises two key functional domains: the N-terminal ATPase domain (NBD, approximately 45 kDa) and the C-terminal substrate-binding domain (SBD, approximately 25 kDa) (32). The N-terminal domain possesses ATP binding and hydrolysis activities, providing the essential energy source for protein conformational changes (33, 34). The C-terminal domain consists of a β-sheet subdomain and an α-helical “lid” region. The former is responsible for recognizing and binding to unfolded or misfolded substrate proteins, while the latter regulates the binding and release of substrates (35). These two domains are connected by a linker (13 amino acids) (36) and function independently in both free and bound states.

The molecular function of HSP70 relies on the allosteric regulation mechanism between the N-terminal and C-terminal domains, which is driven by the conversion of ATP to ADP (37). Through this precise allosteric regulation, HSP70 can effectively facilitate the binding and release of substrates, providing misfolded proteins with the opportunity to correctly refold, thereby fulfilling its molecular chaperone function (38, 39).

### 2.2 Canonical biological functions of HSP70

#### 2.2.1 Regulation of protein homeostasis

As an essential molecular chaperone within cells, HSP70 is widely distributed in the cytoplasm, endoplasmic reticulum, and mitochondria. It assists in the correct folding of nascent polypeptide chains after their release from the ribosome, promoting the folding and formation of the three-dimensional structure of newly synthesized polypeptide chains (11, 12, 38). Under adverse conditions such as heat shock, hypoxia, or oxidative stress, the expression level of HSP70 is significantly upregulated. It maintains cellular homeostasis by repairing misfolded proteins or directing them to the proteasome for degradation.

#### 2.2.2 Regulation of cell survival

HSP70 exerts anti-apoptotic and pro-survival functions through multiple pathways: it can directly bind to apoptotic protease activating factor-1 (Apaf-1), inhibiting the activation of caspase-9 and thus blocking the transmission of apoptotic signals. HSP70 also stabilizes the mitochondrial membrane by preventing the oligomerization of Bax/Bak proteins, effectively inhibiting the release of cytochrome c. Additionally, HSP70 is involved in regulating the balance between autophagy and apoptosis, with mechanisms that may involve inhibiting JNK phosphorylation or interacting with Beclin-1. These multiple protective mechanisms together form the important molecular basis for HSP70 to maintain cell survival.

## 3 Immunoregulatory functions of HSP70

**Figure 1:**
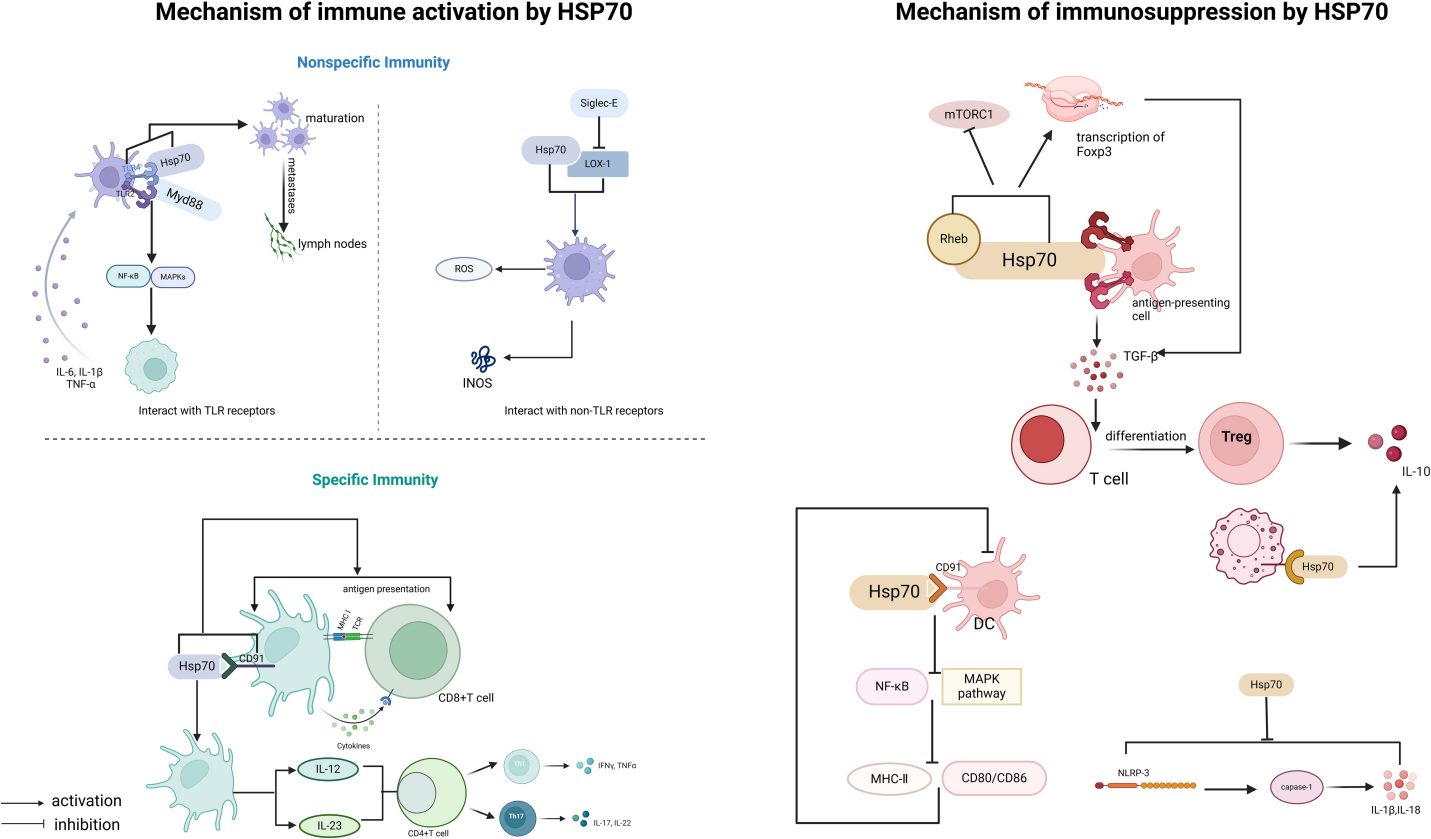
Immunoregulatory functions of HSP70.

HSP70 not only possesses the canonical functions of a molecular chaperone, such as participating in the regulation of protein homeostasis and stress responses, but also plays a complex bidirectional regulatory role in the immune system (**Figure 1**). The dual nature of its immune regulatory function is reflected in two aspects: On the one hand, it enhances immune responses by activating pattern recognition receptors and the antigen presentation system. On the other hand, it maintains immune tolerance by inducing the differentiation of immunosuppressive cells. This dynamic regulatory mechanism makes HSP70 a key molecule linking innate and adaptive immunity.

### 3.1 Immune activation and its mechanisms

The immune-activating function of HSP70 is mainly reflected in two aspects: non-specific immunity and specific immunity (40). Its function is characterized by multidimensionality: In terms of space, it can initiate innate immune responses through cell membrane surface receptors [such as Toll-like receptor 2/4 (TLR2/4)] and also activate specific immunity through the intracellular antigen processing system. In terms of time, it is involved in triggering early inflammatory responses and also affects the formation of late immune memory. The specific mechanisms of action include:

#### 3.1.1 Non-specific immune response

Non-specific immunity is the body’s innate defense system, characterized by broad-spectrum, rapid response, and lack of memory. Its defense mechanisms include physical barriers (such as skin and mucous membranes), chemical barriers (such as lysozyme and stomach acid), immune cells (such as macrophages and natural killer cells), and cytokines.

##### 3.1.1.1 TLR2/4 signaling pathway

Damage-associated molecular patterns (DAMPs) are endogenous molecules released by damaged or dying cells, which can be recognized by the immune system and trigger immune responses (21, 41). DAMPs are mainly recognized by the immune system through two mechanisms: one is through pattern recognition receptors (PRRs) (41), and the other is through inducing the activation of pro-inflammatory cytokines such as IL-18 (42, 43). Studies have found that extracellular HSP70 (eHSP70), as a DAMP, can bind to antigen-presenting cells (such as dendritic cells (DCs) and macrophages) through TLR2 and TLR4 and be recognized by the immune system (44), thereby activating signaling pathways.

This binding can trigger a MyD88-dependent signaling cascade, leading to the activation of nuclear factor-κB (NF-κB) and mitogen-activated protein kinases (MAPKs) (30). The activation of NF-κB further promotes the expression and secretion of pro-inflammatory cytokines (such as tumor necrosis factor-alpha(TNF-α), interleukin-6 (IL-6), and interleukin-1 beta (IL-1β)), amplifying the inflammatory response and enhancing the activation of antigen-presenting cells (APCs) (45). Thus, eHSP70 regulates the inflammatory response through the TLR/MyD88 signaling pathway. Studies have also found that in airway epithelial cells, eHSP70 inhibits the pro-inflammatory ERK1/2-CREB signaling pathway activated by TGF-β1 through the TLR4-GR-DUSP1 signaling pathway (31), reducing the secretion of inflammatory factors. Moreover, the HSP70-TLR4 signaling pathway promotes DC maturation, upregulates the expression of co-stimulatory molecules (such as CD80/86), and promotes the migration of DCs to the lymph nodes (46, 47), thereby bridging non-specific immunity and specific immunity.

##### 3.1.1.2 Interaction with non-TLR receptors

HSP70 can also regulate non-specific immune responses through interactions with Siglec-E and oxidized low-density lipoprotein receptor (LOX-1). The binding of HSP70 to LOX-1 can activate macrophages (48), which produce reactive oxygen species (ROS) during the inflammatory response; meanwhile, macrophages, especially the M1 type, produce a large amount of NO through the expression of inducible nitric oxide synthase (iNOS), enhancing their antimicrobial defense capabilities (49). In contrast, Siglec-E acts as a negative regulator of LOX-1-mediated non-specific immune responses when bound to HSP70 or oxidized LDL. This mechanism helps to activate the immune response, regulate the inflammatory response, and avoid excessive inflammation (50).

#### 3.1.2 Specific immune response

Specific immunity is acquired and mainly mediated by T cells and B cells, characterized by high specificity and memory. According to the cell types mediating the immune response, specific immunity is divided into humoral immunity (mediated by B cells) and cellular immunity (mediated by T cells).

##### 3.1.2.1 Cross-presentation of antigens

Cross-presentation of antigens refers to the process by which antigen-presenting cells (mainly DCs) process exogenous antigens (such as viral or tumor antigens) and present them to CD8+ T cells, thereby activating adaptive immunity. As a molecular chaperone, HSP70 can assist in the folding and stabilization of antigens, promoting their intracellular transport and processing.

CD91 is a multifunctional receptor on the surface of dendritic cells, involved in endocytosis and intercellular signaling (51, 52). Studies have found that the binding of HSP70 to CD91 can promote the internalization of HSP70-antigen complexes by DCs, enhancing antigen processing and presentation efficiency (53). Sec61 is a transmembrane protein complex in the endoplasmic reticulum, mainly responsible for protein translocation and involved in the process of antigens being released from the cytosol into the cytoplasm. HSP70 can interact with Sec61 to promote antigen transport (54), playing a key role in the rocess of cross-antigen presentation.

##### 3.1.2.2 Promotion of Th1/Th17 differentiation

T helper 1 (Th1) and T helper 17 (Th17) cells both originate from naive CD4+ T cells and differentiate into different effector T cell subsets with distinct functions under specific cytokine environments (55, 56). Th1 cells are mainly responsible for mediating cellular immune responses and clearing intracellular pathogens, while Th17 cells mediate pro-inflammatory responses by promoting the mobilization and activation of neutrophils (56).

HSP70 promotes T cell differentiation by regulating the cytokine environment. HSP70-activated DCs secrete IL-12 and IL-23 (57, 58), which induce the differentiation of Th1 and Th17 cells, respectively (59). Th1 cells secrete interferon-gamma (IFN-γ) to enhance the activity of cytotoxic T cells (CTLs), while Th17 cells recruit neutrophils through IL-17, working together to clear pathogens.

### 3.2 Immune suppression and its mechanisms

HSP70 not only plays a role in immune activation but also acts as an important regulator in immune suppression. Its immune-suppressive effects are mainly reflected in the induction of regulatory immune cells and the regulation of anti-inflammatory signaling pathways.

#### 3.2.1 Promotion of regulatory T cell differentiation and inhibition of dendritic cell maturation

Regulatory T cells (Tregs) play a key role in immune tolerance and the suppression of excessive immune responses. Studies have found that HSP70 can bind to TLR2/TLR4 on the surface of antigen-presenting cells (APCs) (44, 60), inducing APCs to secrete TGF-β, which in turn promotes the differentiation of naive T cells into Tregs (61). Rheb is an upstream activator of mTORC1, and HSP70 can bind to Rheb and induce its degradation, thereby inhibiting the activity of mTORC1 (62) and enhancing Foxp3 transcription (63). Foxp3 can inhibit the production of pro-inflammatory cytokines while promoting the secretion of immune-suppressive cytokines (such as interleukin-10 (IL-10) and TGF-β), maintaining the immune-suppressive function of Treg cells and thus sustaining an immune-suppressive microenvironment. Additionally, extracellular HSP70 (eHSP70) binds to the CD91 receptor on the surface of DCs (53), inhibiting the NF-κB and MAPK pathways, downregulating the expression of co-stimulatory molecules (CD80/86) and major histocompatibility complex class II (MHC-II), leading to DC dysfunction and weakened antigen-presenting capabilities (64).

#### 3.2.2 Regulation of anti-inflammatory signaling pathways

##### 3.2.2.1 Activation of IL-10 secretion

IL-10 is an important anti-inflammatory factor that can inhibit the production of pro-inflammatory cytokines and suppress the function of antigen-presenting cells. Studies have found that HSP70 in MSCs-Exo can reduce the inflammatory response after liver transplantation and improve liver function by activating IL-10 secretion (65). HSP70 can bind to receptors on the surface of macrophages, activate downstream signaling pathways, and promote IL-10 secretion (66), helping to maintain the balance of the immune system and prevent excessive immune responses from causing damage to the body.

##### 3.2.2.2 Inhibition of NLRP3 inflammasome

The NLRP3 inflammasome is an important component of the non-specific immune system, capable of activating caspase-1 (interleukin-1β-converting enzyme), promoting the maturation and release of IL-1β and IL-18 (67), and triggering a strong inflammatory response. HSP70 can inhibit the NLRP3 inflammasome through several mechanisms (68–70). From the study, it is evident that the absence of HSP70 exacerbates NLRP3-dependent peritonitis and enhances caspase-1 activation and IL-1β production in bone marrow-derived macrophages (BMDMs) of mice, while also increasing the number and size of ASC/NLRP3 specks (68). Conversely, overexpression of HSP70 inhibits these processes. HSP70 can also directly interact with NLRP3, and this interaction disappears upon NLRP3 inflammasome activation (20, 68), suggesting that HSP70 may inhibit NLRP3 inflammasome formation by binding to NLRP3 and altering its conformation. Additionally, HSP70 significantly reduces the LPS (lipopolysaccharide)-induced elevation of NLRP3 protein levels, thereby suppressing the formation and activation of the NLRP3 inflammasome (20).

Upon comprehensively analyzing the roles of HSP70 in immune activation and immune suppression, we found that HSP70 binding to the same type of receptor on the same kind of cell may induce different cytokines and even exert completely opposite effects on immune regulation. For example, HSP70 binding to TLR2/TLR4 on antigen-presenting cells (APCs) can trigger the MyD88 cascade to activate the immune response (71), as well as induce APCs to secrete TGF-β (61), which promotes the differentiation of naive T cells into regulatory T cells (Tregs) to suppress the immune response. Similarly, HSP70 binding to the CD91 receptor can enhance antigen presentation capacity, but it can also weaken this capacity by inhibiting the NF-κB and MAPK pathways. Its effects are highly dependent on factors such as co-stimulatory signals in the microenvironment (e.g., cytokine concentrations, oxidative stress levels).

## 4 The dual regulatory role of HSP70 in tumor immunity

HSP70 can not only promote tumor progression by facilitating cancer cell proliferation, metastasis, and drug resistance but also enhance the body’s anti-tumor immune response by activating immune cells and mediating immune responses (72, 73).

### 4.1 Pro-tumor mechanisms of HSP70

#### 4.1.1 Direct pro-cancer effects

Studies have shown that HSP70 is highly expressed in various tumor cells and exerts pro-cancer effects by inhibiting apoptosis (74). HSP70 exerts its anti-apoptotic effects by regulating key effector molecules in apoptotic signaling pathways.

In the intrinsic apoptosis pathway, the HSP70-CHIP complex can promote the proteasomal degradation of apoptosis signal-regulating kinase 1 (ASK1), thereby inhibiting the activation of c-Jun N-terminal kinase (JNK) and p38 (75). Notably, the activation of JNK is crucial for the release of cytochrome C from mitochondria and the initiation of the endogenous apoptotic pathway (76). HSP70 can also inhibit apoptosis through the following mechanisms: ① inhibiting the translocation of pro-apoptotic protein Bax to the mitochondrial outer membrane and preventing Bax oligomerization to form pores, thereby inhibiting the release of cytochrome C and other apoptotic factors from mitochondria to the cytoplasm (77); ② inhibiting Bcl-2 transcription and blocking Bax translocation to mitochondria, thus preventing an increase in mitochondrial outer membrane permeability (78, 79); ③ binding to the CARD domain of Apaf-1, preventing the recruitment of caspase-9 to the apoptosome (73).

**Figure 2:**
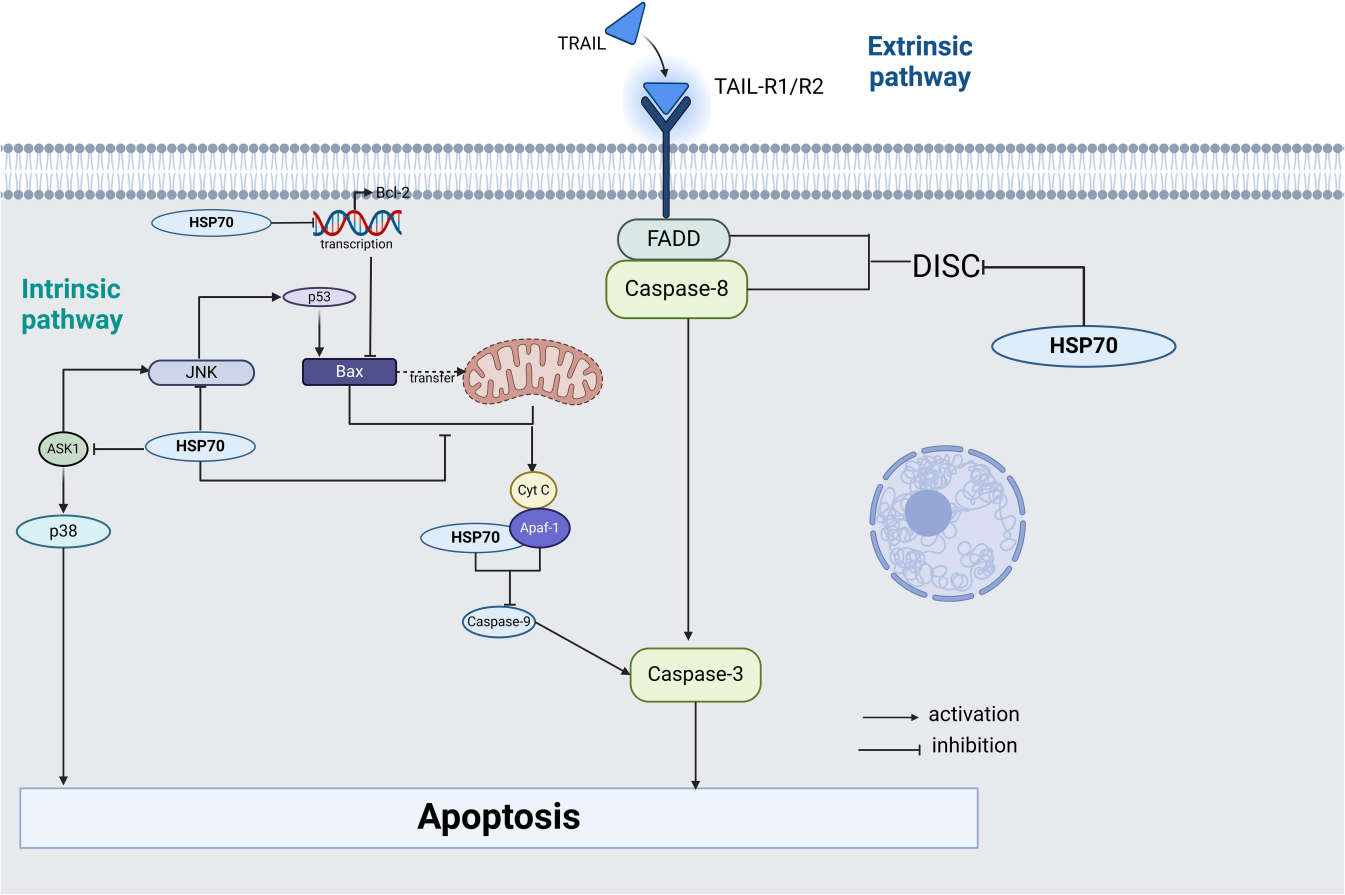
Key effector molecules regulated by HSP70 in intrinsic and extrinsic apoptosis signaling pathways.

In the extrinsic apoptotic pathway, HSP70 exerts anti-apoptotic effects through the following mechanisms: ① interacting with TNF-related apoptosis-inducing ligand receptors 1 (TRAIL-R1) and TRAIL-R2, blocking the formation of the death-inducing signaling complex (DISC); ② forming a complex with FANCC protein, inhibiting the activation of the extrinsic apoptotic pathway (80, 81); ③ in BCR-ABL expressing cells, binding to death receptors DR4 and DR5, inhibiting the formation of apoptotic signaling complexes (82). Additionally, HSP70 can also inhibit the activation of JNK and p38 induced by DNA damage (83), which play key roles in apoptotic signaling (**Figure 2**).

The epithelial-mesenchymal transition (EMT) is an important process for tumor cells to acquire invasive and metastatic capabilities, characterized by changes in cell morphology, reduced cell adhesion, and enhanced migration and invasion abilities. It has been found that HSP70 overexpression can promote the EMT process and tumor metastasis by activating signaling pathways such as NF-κB and upregulating the expression of mesenchymal markers like N-cadherin (84).

The interaction between HSP70 and vascular endothelial growth factor (VEGF) is also noteworthy. VEGF, a member of the platelet-derived growth factor family, plays a crucial role in tumor angiogenesis and vascular permeability regulation. VEGF is highly expressed in the tumor microenvironment, promoting tumor angiogenesis to provide nutrients and oxygen to tumors, thereby facilitating tumor growth and metastasis.

Clinical studies have found that the levels of HSP70 and VEGF in the serum of pancreatic cancer patients are significantly increased (85), indicating that HSP70 may promote VEGF expression through specific mechanisms.In hepatocellular carcinoma cells HepG2, extracellular HSP70/HSP70-PCs can upregulate VEGF expression through the HIF-1α (hypoxia-inducible factor-1α) signaling pathway. However, in some cases, HSP70 may have the opposite effect. For example, in osteoblasts, HSP70 negatively regulates VEGF synthesis by inhibiting p38 MAPK phosphorylation (86). From a cellular functional perspective, tumor cells typically reside in a hypoxic and nutrient-deprived tumor microenvironment, which drives HSP70 to promote angiogenesis to support tumor cell proliferation, invasion, and metastasis. Specifically, HSP70 stabilizes hypoxia-inducible factor-1α (HIF-1α), preventing its degradation via the ubiquitin-proteasome pathway, thereby facilitating the direct binding of HIF-1α to the VEGF promoter, which drives VEGF transcription and expression, ultimately enhancing angiogenesis. In contrast, in osteoblasts, due to the demands of mechanical stress and bone homeostasis maintenance, HSP70 tends to suppress angiogenesis to prioritize bone mineralization and repair processes. In osteoblasts, HSP70 negatively regulates VEGF synthesis by inhibiting the activation of p38 MAP kinase. Based on these findings, we hypothesize that the differential regulatory effects of HSP70 on VEGF may stem from the distinct microenvironments, functional requirements, and signaling pathways mediated by different cell types.

Therefore, the regulatory role of HSP70 in VEGF expression exhibits significant microenvironment dependency, and its specific mechanisms require in-depth analysis based on specific experimental conditions, disease contexts, and research objectives. However, current research on HSP70-mediated VEGF regulation in non-tumor cells remains limited. Future studies are needed to further explore the diverse functions of HSP70 across different cell types and physiological or pathological conditions, to comprehensively elucidate its regulatory networks, and to provide new theoretical foundations and potential therapeutic targets for precision medicine in related diseases.

#### 4.1.2 Molecular mechanisms promoting tumor immune escape

Tumor immune escape is a key process in the development of malignant tumors, essentially involving tumor cells evading recognition and elimination by the host immune system through various molecular mechanisms, thereby allowing continuous growth and spread within the host. This complex process involves multiple immune checkpoints, where HSP70 plays a significant role.

The tumor microenvironment (TME) is a complex ecosystem, including the extracellular matrix, immune cells, fibroblasts, vascular endothelial cells, and various cytokines and metabolic products (87, 88). In this unique environment, HSP70 can promote immune escape through multiple mechanisms, such as inhibiting the activity and function of T cells and natural killer (NK) cells; HSP70 can also affect the maturation of dendritic cells (DCs), interfering with their antigen-presenting functions, thereby reducing the activation efficiency of T cells (73).

In the tumor microenvironment, myeloid-derived suppressor cells (MDSCs) are a group of myeloid cells with immune-suppressive functions. HSP70 can promote immune escape by interacting with MDSCs. Studies have found that 5-fluorouracil (5-FU) can selectively eliminate MDSCs while activating the NLRP3 inflammasome (89). This process is accompanied by the activation of caspase-1 and the production of IL-1β, ultimately exerting anti-tumor effects (89). Research has shown that HSP70 on the surface of exosomes can interact with TLR2 on MDSCs, thereby activating the immune-suppressive functions of MDSCs (90). The mechanisms are as follows. After tumor - derived exosomal HSP70 binds to TLR2 on the surface of MDSCs, it activates STAT3 phosphorylation, induces the expression of arginase 1 (Arg1) and inducible nitric oxide synthase (iNOS), and enhances the immunosuppressive activity of MDSCs (90). Meanwhile, the HSP70 - TLR2 signal can also promote the secretion of IL - 10 through the ROS - dependent ERK1/2 pathway, further inhibiting the function of CD8+ T cells. The tumor microenvironment is often in a hypoxic and acidic state. Based on the activation of the STAT3 pathway by HSP70 - exosomes through the TLR2 - MDSCs axis (91), it may further amplify the immunosuppressive effect mediated by HSP70 by regulating the membrane localization or signal transduction efficiency of TLR2.

#### 4.1.3 Regulation of the PD-1/PD-L1 axis

The programmed cell death protein 1/programmed death-ligand 1 (PD-1/PD-L1) signaling pathway, as a key immune checkpoint, plays a core role in maintaining immune homeostasis and tumor immune escape. HSP70 participates in the regulation of this pathway through various molecular mechanisms:

1. Epigenetic regulation: HSP70 can enhance histone acetyltransferase (HAT) activity, promoting the histone H3K27ac modification in the PD-L1 promoter region, thereby upregulating PD-L1 transcription.
2. Signal transduction pathway: HSP70 binding to TLR4 activates the NF-κB pathway (45), inducing the co-expression of PD-L1 and IL-10, forming an immune-suppressive microenvironment (92).
3. Protein stability regulation: As a molecular chaperone, HSP70 can stabilize PD-L1 protein, preventing its degradation, thereby increasing PD-L1 expression on the cell surface (73).

However, Hsc70, a member of the HSP70 family, plays an opposing regulatory role. It can promote the lysosomal degradation of PD-L1 by competitively binding to it, thereby reducing the expression of PD-L1 on the cell membrane (93). Specifically, Hsc70 targets PD-L1 for lysosomal degradation through the endosomal microautophagy (eMI) pathway and competitively inhibits CMTM6-mediated recycling of PD-L1 (93).

Although Hsc70 and Hsp70 exhibit only minor differences in amino acid sequences, they display significant distinctions in expression patterns, subcellular localization, functions, and regulatory mechanisms. Firstly, Hsc70 is constitutively expressed, whereas the expression of Hsp70 is typically induced under stress conditions. Hsc70 is primarily localized in the cytoplasm but can shuttle between the cytoplasm and the nucleus under specific conditions, participating in the regulation of nucleocytoplasmic transport. In contrast, Hsp70 predominantly functions as a molecular chaperone under stress conditions.

In terms of function, Hsc70 is not only involved in classical chaperone activities but also plays a critical role in protein degradation, particularly by facilitating the degradation of specific proteins through chaperone-mediated autophagy (CMA) and endosome-associated microautophagy (EmiA) (94, 95). Additionally, Hsc70 protects the integrity of lysosomal membranes (96). In comparison, Hsp70 is more inclined to stabilize proteins or regulate their functions to support cell survival and proliferation.

Moreover, Hsc70 exhibits unique characteristics in secretion and plasma membrane localization. Its secreted form can be induced by contact inhibition or serum deprivation, and this secreted form has been shown to inhibit cell proliferation and promote contact inhibition (97), a feature that Hsp70 does not possess. Regarding immune regulation, both Hsc70 and Hsp70 can be released into the extracellular space during viral infections or upon cytokine stimulation. These differences enable Hsc70 and Hsp70 to play both overlapping and distinct roles in maintaining cellular homeostasis and contributing to disease pathogenesis (97).

#### 4.1.4 Activation mechanism of the MerTK signaling pathway

The MerTK signaling pathway, as an important cell signaling system, plays a key role in regulating apoptosis, phagocytosis, and immune regulation. In the tumor microenvironment, the expression level of MerTK is closely related to immune escape, mainly involving the functional regulation of tumor-associated macrophages (TAMs). Studies have shown that MerTK-positive TAMs typically exhibit significant immune-suppressive characteristics, inhibiting T cell function by expressing immune checkpoint molecules such as PD-L1 (98), thereby promoting tumor immune escape. This finding makes MerTK a potential target for cancer therapy.

Recent studies have found that HSP70 secreted by tumor cells can bind to TLR2 on the surface of macrophages, forming an HSP70-TLR2 complex, which upregulates MerTK expression and promotes the polarization of macrophages to the M2 type (66). This process further enhances the immune-suppressive characteristics of the tumor microenvironment. Therefore, intervention in the HSP70-TLR2-MerTK signaling pathway may provide new strategies for cancer treatment.

### 4.2 Anti-tumor effects of HSP70

The anti-tumor effects of HSP70 in tumor immunity are mainly reflected in its ability to activate the immune system and enhance the body’s immune surveillance and killing capabilities against tumors.

#### 4.2.1 Regulation of immunogenic cell death

Immunogenic cell death is a unique form of cell death that can trigger specific immune responses in hosts with normal immune functions. As an important member of damage-associated patterns, HSP70 plays a key role in the ICD process. When tumor cells undergo ICD, the released HSP70 can activate APCs and enhance their antigen-presenting capabilities. By promoting ICD, HSP70 can significantly increase the immunogenicity of tumor cells, thereby enhancing the anti-tumor immune response.

#### 4.2.2 Activation of NK cells by exosomal HSP70

Tumor-derived exosomes are rich in HSP70, and these exosomes can significantly induce the body’s anti-tumor immune response (99). Studies have found that HSP70 can selectively activate NK cells by binding to receptors on the surface of NK cells. Notably, exosomes or tumor cell lysates lacking HSP70 do not possess this activation capability (100). Further research has indicated that TREM-1 (Triggering Receptor Expressed on Myeloid cells-1) receptors expressed on the surface of myeloid cells and NK cells may be involved in this process (101), but the specific molecular mechanisms still need to be further clarified.

#### 4.2.3 Mediation of specific anti-tumor immune responses

HSP70 can form stable complexes with tumor antigen peptides. These complexes are internalized by DCs through the CD91 receptor on their surface and then transported to the cytoplasm via the endosomal escape mechanism. In the cytoplasm, these antigen peptides are processed by proteasomes and bind to MHC-I molecules, ultimately activating CD8+ T cells and mediating specific anti-tumor immune responses. This mechanism has been verified in melanoma and lung cancer models, confirming that HSP70-antigen complexes can elicit a strong anti-tumor immune response (102).

Current research findings indicate that HSP70 exhibits a complex bidirectional regulatory effect on immune-related proteins, such as its bidirectional regulation of VEGF and the PD-1/PD-L1 axis. This regulatory characteristic may be dynamically regulated by epigenetic modifications in the tumor microenvironment, suggesting that the pro-cancer or anti-cancer effects of HSP70 are environment-dependent. This discovery provides a new perspective for a deeper understanding of the role of HSP70 in tumor immunity and also presents challenges for the development of precision treatment strategies based on HSP70.

## 5 Therapeutic strategies and future prospects

**Figure 3:**
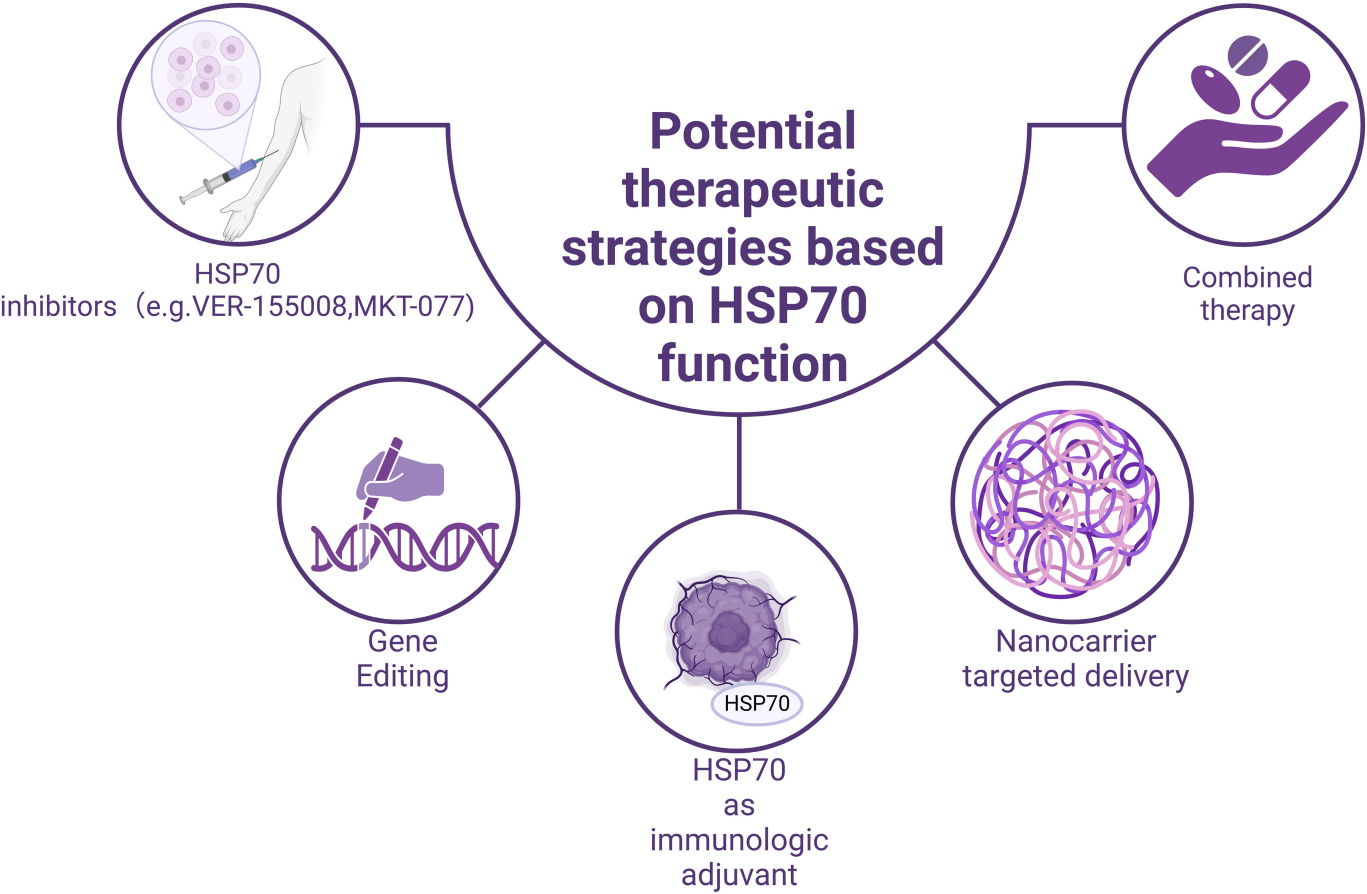
Potential therapeutic strategies based on the functions of HSP70.

Based on the dual role of HSP70 in immune regulation, researchers have developed various potential therapeutic strategies, providing new ideas and directions for tumor treatment (**Figure 3**).

### 5.1 Development and application of HSP70 inhibitors

Given that HSP70 mediates tumor immune evasion in the tumor microenvironment through mechanisms such as promoting the differentiation of Treg cells, the development of specific HSP70 inhibitors may help restore the body’s anti-tumor capabilities. Several small-molecule inhibitors have shown promising application prospects (**Table 1**). For example,VER-155008, a highly selective HSP70 inhibitor, exerts its anti-tumor function by competitively binding to the ATP-binding site of HSP70 (114–117). In experiments related to pheochromocytoma, VER-155008 was found to significantly inhibit the proliferation of PC12 cells (a cell line derived from rat adrenal pheochromocytoma, PHEO) *in vitro*. After two weeks of treatment, the tumor volume was markedly reduced from 2584.7 ± 525.6 mm³ to 1253.9 ± 157.8 mm³ (P < 0.001) (103); Another representative inhibitor, MKT-077, a cationic rhodamine dye (118), regulates tumor cell proliferation by inhibiting the chaperone function of the HSP70 family (119, 120). The study found that the concentration of MKT-077 was positively correlated with its inhibitory effect on tumor cells. When the concentration of MKT-077 reached 2 µg/ml or higher, it exhibited significant cytotoxicity against multiple tumor types (121), for example, MKT-077 selectively accumulates in mitochondria within medullary thyroid carcinoma (MTC) cells, disrupts mitochondrial activity, induces oxidative stress and apoptosis, thereby inhibiting cell proliferation and survival. Experimental data show that after 48 hours of MKT-077 treatment, the survival rate of TT cells significantly decreased, with an IC50 value of 0.74 μm, indicating its potent cytotoxicity against MTC cells (104, 105). Besides, in estrogen receptor-positive breast cancer, S1g-2, acting as a specific inhibitor of the Hsp70-Bim protein-protein interaction, can overcome tamoxifen resistance. Studies using xenograft models have shown that disrupting the Hsp70-Bim interaction with S1g-2 can reduce tumor volume by approximately threefold (106). Furthermore, experiments have revealed that S1g-2 can reverse the specific protective effect of Hsp70-Bim protein-protein interaction (PPI) in chronic myeloid leukemia (CML). S1g-2 exhibits 5-10 times higher apoptosis-inducing activity in CML cells compared to other cancer cells, normal lymphocytes, and BaF3 cells (107, 108).

**Table 1:** Preclinical and clinical trials of HSP70-related therapies.

Therapy	Name	Drug Development Stages	NCT number	Clinical phase	Condition	References
HSP70 inhibitor	VER-155008	Preclinical			pheochromocytoma	Xu F, 2019 (103)
HSP70 inhibitor	MKT-077	Preclinical			multiple tumor types	Hong S-K,2022 Starenki D,2015 (104, 105)
Inhibitor of the Hsp70-Bim protein-protein interaction	S1g-2	Preclinical			estrogen receptor-positive breast cancer, CML	Song T,2024 Song T,2021 Zhang H,2022 (106–108)
HSP70 inhibitor	Minnelide	Clinical	NCT04896073	Phase II	Advanced gastrointestinal tumors	Skorupan N, 2022 (109)
Vaccines	AFP-HSP70	Preclinical			Hepatocellular carcinoma	Lan Y-H,2007 (110)
Vaccines	Autologous tumor-derived HSP70 protein vaccine	Clinical	NCT00027144	Phase I	Chronic myeloid leukemia	Zihai Li,2005 (111)
Vaccines	Leukocyte-derived HSP70-peptide complexes	Clinical	NCT00030303	Phase I	Chronic myeloid leukemia	Li Z, 2005 (112)
Vaccines	Tyrosinase and gp100 peptides fused with OVA BiP peptide and recombinant HSP70 protein	Clinical	NCT00005633	Phase I	Stage III or stage IV melanoma	Philip O. Livingston,2013 (113)

Moreover, VER-155008 does not inhibit HSP90, which is advantageous for specifically targeting HSP70 without affecting the normal functions of HSP90. This specificity allows the drug to precisely interfere with the survival mechanisms of tumor cells while minimizing interference with normal cells, thereby enhancing the precision and efficacy of treatment. Additionally, since HSP90 plays important physiological roles in normal cells, inhibiting it could lead to toxicity in these cells. The lack of effect of VER-155008 on HSP90 reduces toxicity to normal cells, improves treatment safety, and minimizes potential side effects.

### 5.2 Potential of HSP70 as an immunological adjuvant

The unique role of HSP70 in antigen presentation and immune activation makes it a promising candidate for an immunological adjuvant. Its mechanism primarily involves forming HSP70-antigen complexes, which promote cross-presentation of antigens and subsequently activate CD8+ T cells (122). In melanoma and lung cancer models, HSP70-antigen complexes have shown good anti-tumor effects. A previous study constructed a therapeutic peptide vaccine, HSP70-P/AFP-P, by linking the functional peptide of heat shock protein 70 (HSP70) with an epitope peptide of alpha-fetoprotein (AFP) to enhance the immune response against AFP-expressing tumors. In a BALB/c mouse model, the HSP70-P/AFP-P vaccine significantly increased the number of AFP-specific CD8+ T cells. The HSP70-P/AFP-P vaccine significantly prolonged survival, with vaccinated mice surviving for over 60 days, whereas all mice in the control group died within 35 days (123), Studies have also found that mice vaccinated with pcDNA3.1-AFP-HSP70 exhibited significantly higher cytotoxic T lymphocyte (CTL) activity compared to the control group. Moreover, CTL activity increased markedly with the rise in the effector-to-target cell ratio (E/T) (P < 0.001) (110).These findings provide a theoretical basis for developing novel cancer vaccines.

However, clinical research and trials on HSP70 inhibitors and vaccines are scarce at present. Current clinical trials have found that Minnelide, a water-soluble pro-drug derivative of Triptolide, exerts its anti-tumor effects by suppressing the expression of HSP70 (124). Minnelide has demonstrated significant anti-tumor efficacy in various cancers (125, 126). In preclinical studies, Minnelide markedly suppressed tumor growth and metastasis (127–129), and it has also shown potential therapeutic effects in clinical trials targeting advanced refractory adenosquamous carcinoma of the pancreas (109); Compared to HSP70 inhibitors, HSP70 vaccines have relatively more clinical trials (**Table 1**). For example, the autologous tumor-derived HSP70 protein vaccine and the leukocyte-derived HSP70-peptide complexes have demonstrated favorable tolerability and potential immunotherapeutic efficacy in clinical trials involving patients with chronic myeloid leukemia (CML) (112). Besides, Tyrosinase and gp100 peptides fused with OVA BiP peptide and recombinant HSP70 protein have also demonstrated promising therapeutic potential in melanoma. It is expected that more clinical researchers will explore its application value in clinical treatment in the future.

### 5.3 Gene editing therapy based on HSP70

With the development of gene-editing technologies such as CRISPR-Cas9, therapeutic strategies targeting the HSP70 gene have shown great potential. Given the close association between the high expression of HSP70 in tumor cells and their anti-apoptotic characteristics, precise regulation of HSP70 expression through gene-editing techniques can significantly enhance the sensitivity of tumor cells to apoptosis. Moreover, combining gene-editing technologies with other treatments, such as magnetic hyperthermia, can further improve therapeutic outcomes while reducing damage to normal tissues, offering a new precision medicine approach for cancer treatment. Recently, a research team developed a magnetothermal-activated CRISPR-Cas9 gene-editing system to target the HSP70 and BCL2 genes in tumor cells. This system, based on a magnetothermal nanoparticle platform, utilizes the mild thermal effect (42°C) generated by an alternating magnetic field to activate the CRISPR-Cas9 system, thereby precisely targeting the HSP70 and BCL2 genes and significantly enhancing tumor cell apoptosis (130).

### 5.4 Application of nanotargeted delivery systems

The rapid development of nanotechnology provides new solutions for the precise delivery of HSP70 inhibitors. By designing nanotargeted delivery systems, the specific enrichment of HSP70 inhibitors at tumor sites can be achieved, thereby improving drug therapeutic effects and reducing systemic toxicity. These delivery systems can not only enhance drug bioavailability but also achieve further improvements in targeting through surface modifications. Additionally, nanotechnology can be used for the delivery of HSP70-antigen complexes, enhancing antigen presentation efficiency and immune activation effects.

### 5.5 Exploration of combined treatment strategies

The combined application of HSP70 inhibitors with other treatment methods shows significant synergistic effects. For example, when VER - 155008 and anti - PD - 1 antibody are used separately, drug resistance or side effect may occur (131), leading to a decline in efficacy. However, if the two are used in combination, the anti - tumor immune response may be significantly enhanced (131), and this combination may even be superior to single - agent therapies in terms of controlling tumor volume and weight. Moreover, the combination of HSP70 inhibitors with chemotherapeutic drugs also shows promising prospects, improving chemotherapy effects by inhibiting tumor cell drug resistance and promoting apoptosis. Notably, the synergistic effect of dual inhibition of HSP70 and autophagy with cisplatin treatment can significantly reduce tumor cell metabolic activity and growth (132). These combined treatment strategies not only improve therapeutic effects but also effectively reduce the side effects of monotherapy, providing new ideas for comprehensive cancer treatment.

### 5.6 Challenges in targeting HSP70 for therapeutic applications

Although therapies targeting HSP70 hold great promise, they face significant challenges in clinical translation, which may be mainly attributed to the inefficient drug delivery system, complex interactions within the tumor microenvironment, and the emergence of drug resistance mechanisms.

The abnormal vasculature and dense extracellular matrix in the tumor microenvironment severely impede the uniform distribution of HSP70 - targeted drugs, potentially resulting in suboptimal drug concentrations in the tumor area. Although current nanotechnology - based delivery systems (Section 5.4) have shown promising potential, further optimization is required to enhance tumor penetration and minimize off - target effects. Additionally, although HSP70 is overexpressed in many cancers, its basal expression in normal cells (especially under stress conditions) has a great impact on treatment specificity. For example, cardiomyocytes and neurons rely on HSP70 for stress adaptation, and its inhibition may exacerbate tissue damage. In this context, methods such as utilizing tumor - specific post - translational modifications or altering microenvironmental cues (such as the hypoxic environment) can be considered to improve selectivity.

Furthermore, the problem of drug resistance to HSP70 - targeted therapy also affects the treatment effect. This drug resistance may be attributed to multiple complex mechanisms: First, when HSP70 is inhibited, tumor cells may compensatorily upregulate the expression of other molecular chaperones (such as HSP90, HSP60, etc.) to maintain protein homeostasis and cell survival. This compensatory mechanism enables tumor cells to continue to survive and proliferate under the action of HSP70 - targeted drugs. Second, the intracellular localization of HSP70 has an important impact on its function. HSP70 - targeted therapy may change its subcellular localization, causing it to transfer to other cell regions under the action of drugs, thus evading drug inhibition. Tumor heterogeneity is also an important source of drug resistance to HSP70 - targeted therapy. Tumor cells show significant heterogeneity at the genetic and epigenetic levels, resulting in different sensitivities of different cells to HSP70 - targeted drugs. During the treatment process, tumor cell subsets with stronger drug resistance may gradually accumulate, forming drug tolerance. These drug resistance mechanisms make HSP70 - targeted drugs may show certain efficacy in the initial treatment, but over time, tumor cells may gradually adapt and develop drug resistance, leading to treatment failure. Consider combining HSP70 inhibitors with inhibitors of other molecular chaperones to reduce the possibility of tumor cells developing drug resistance through compensatory mechanisms. We can also deeply analyze the genetic and epigenetic characteristics of each patient’s tumor, understand its HSP70 expression level, the activation status of related signaling pathways, and the characteristics of the tumor microenvironment, and tailor the most effective treatment plan for the patient. This personalized treatment can not only improve the success rate of treatment but also reduce unnecessary drug side effects.

## 6 Challenges and future directions in HSP70 research

Despite the promising therapeutic potential of HSP70 in cancer treatment, several significant challenges remain to be addressed to fully exploit its role. First, the precision of targeted therapy is critical. It is essential to ensure that drugs specifically inhibit the function of HSP70 in tumor cells without affecting its normal physiological roles in healthy cells, thereby minimizing toxicity and side effects.

Second, the complexity of the tumor microenvironment poses another challenge. The heterogeneous expression of HSP70 in different tumor cells and the presence of various immune cells within the tumor microenvironment can influence treatment efficacy. Therefore, a deeper understanding of the microenvironmental factors that regulate HSP70 expression and function is necessary to design more effective therapeutic strategies.

Moreover, combination therapies based on HSP70, such as those integrating immunotherapy or chemotherapy, require careful consideration and thorough preclinical evaluation to optimize treatment protocols and elucidate their underlying mechanisms. Clinical trials are also needed to validate the safety and efficacy of these combination therapies in patients.

The immunomodulatory functions of HSP70 extend beyond the realm of oncology, and its potential roles in non-neoplastic diseases warrant further exploration. In autoimmune diseases, HSP70 may regulate excessive immune responses through mechanisms such as inducing regulatory T cell (Treg) differentiation or suppressing NLRP3 inflammasome activation. In chronic inflammatory diseases, its anti-inflammatory properties and protein homeostasis maintenance functions could serve as potential therapeutic targets. Future research should systematically elucidate the immunoregulatory networks of HSP70 across diverse pathological contexts, thereby expanding its therapeutic potential in immune-related disorders.

Furthermore, HSP70 holds significant promise as a predictive biomarker for immunotherapy responses. Future studies should employ multi-omics analyses and prospective cohort investigations to establish the correlation between dynamic HSP70 expression patterns and patient outcomes, while exploring its feasibility as a treatment response biomarker. For instance, monitoring changes in serum or exosomal HSP70 levels before and after treatment may provide valuable insights for optimizing individualized therapeutic strategies.

In summary, HSP70 functions as a “double-edged sword” in immunomodulation, with its functional complexity presenting both challenges and opportunities. Through interdisciplinary collaboration, technological innovation, and the deepening of translational research, we may unlock the multidimensional value of HSP70 in precision medicine, thereby opening new avenues for the treatment of cancer and other immune-related diseases.
